# Identification of Oxygen-Responsive Transcripts in the Silage Inoculant *Lactobacillus buchneri* CD034 by RNA Sequencing

**DOI:** 10.1371/journal.pone.0134149

**Published:** 2015-07-31

**Authors:** Felix Gregor Eikmeyer, Stefan Heinl, Hans Marx, Alfred Pühler, Reingard Grabherr, Andreas Schlüter

**Affiliations:** 1 Institute for Genome Research and Systems Biology, Center for Biotechnology, Bielefeld University, Bielefeld, Germany; 2 CD Laboratory for Genetically Engineered Lactic Acid Bacteria, University of Natural Resources and Life Sciences, Vienna, Austria; 3 CD Laboratory for Biotechnology of Glycerol, University of Natural Resources and Life Sciences, Vienna, Austria; 4 Department of Biotechnology, BOKU-VIBT University of Natural Resources and Life Sciences, Vienna, Austria; Université Laval, CANADA

## Abstract

The *Lactobacillus buchneri* CD034 strain, known to improve the ensiling process of green fodder and the quality of the silage itself was transcriptionally analyzed by sequencing of transcriptomes isolated under anaerobic *vs*. aerobic conditions. *L*. *buchneri* CD034 was first cultivated under anaerobic conditions and then shifted to aerobic conditions by aeration with 21% oxygen. Cultivations already showed that oxygen was consumed by *L*. *buchneri* CD034 after aeration of the culture while growth of *L*. *buchneri* CD034 was still observed. RNA sequencing data revealed that irrespective of the oxygen status of the culture, the most abundantly transcribed genes are required for basic cell functions such as protein biosynthesis, energy metabolism and lactic acid fermentation. Under aerobic conditions, 283 genes were found to be transcriptionally up-regulated while 198 genes were found to be down-regulated (p-value < 0.01). Up-regulated genes i. a. play a role in oxygen consumption *via* oxidation of pyruvate or lactate (*pox*, *lctO*). Additionally, genes encoding proteins required for decomposition of reactive oxygen species (ROS) such as glutathione reductase or NADH peroxidase were also found to be up-regulated. Genes related to pH homeostasis and redox potential balance were found to be down-regulated under aerobic conditions. Overall, genes required for lactic acid fermentation were hardly affected by the growth conditions applied. Genes identified to be differentially transcribed depending on the aeration status of the culture are suggested to specify the favorable performance of the strain in silage formation.

## Introduction

Lactobacilli are present in a broad range of habitats and are applied in a variety of processes to produce and conserve food such as dairy (yoghurt, cheese) and meat products as well as feed, e. g. silage [1]. Fermentations driven by lactobacilli benefit from lactic acid production, which is a characteristic feature of these kind of microorganisms. In the course of lactic acid fermentations, growth of spoiling microorganisms is prevented due to the production of lactic acid and other short-chain fatty acids. Additionally, lactobacilli are directly used as probiotics for human and animal nutrition [1]. Since most *Lactobacillus* species are aerotolerant obligate fermentative bacteria, many production processes or growth conditions are characterized by anaerobic or microaerobic conditions. Some studies analyzed the influence of oxygen on some *Lactobacillus* species. It was observed that aeration of *Lactobacillus* cultures generally leads to the formation of increased amounts of acetate [2,3] probably because of pyruvate oxidase activity which was described for *L*. *plantarum* strains Lp80 and WCFS1 [4,5]. For *L*. *plantarum* WCFS1 it was also shown by a proteomic approach that the NADH peroxidase is involved in hydrogen peroxide detoxification [5].

Lactic acid fermentation is also the key pathway of lactobacilli required for ensiling of fresh fodder. Under adequate conditions, ensiling occurs by epiphytic lactic acid bacteria (LAB) [6]. During the ensiling process, LAB are exposed to changing gas phase compositions. First, aerobic conditions persist during the harvesting and preparation of the green fodder. Ensiling begins after the ensiled material is stored under conditions preventing aeration. During the fermentation water soluble carbohydrates are consumed and lactic acid is produced leading to decreasing pH values by LAB. When the silage is opened, oxygen again gains access to the ensiled material. Aerobic conditions may then promote growth of spoiling microorganisms such as yeast, fungi and other bacteria [7].

To enhance a stable ensiling process, some LAB strains are applied as inoculum to the fresh plant biomass before ensiling. *Lactobacillus buchneri* strains were shown to be efficient inoculants for grass silage resulting in an improved stability upon oxygen exposure of the conserved plant material [8–10]. Its mode of action is attributed to its heterofermentative metabolism and the production of higher levels of acetate [11].

The genome sequence of *L*. *buchneri* CD034 has recently been established and interpreted [12] as a basis to uncover genetic determinants that are of importance for *L*. *buchneri* applications in ensiling. Genome sequencing showed that *L*. *buchneri* CD034 possesses a circular chromosome and three additional plasmids. Annotation of the genome revealed the absence of genes encoding fructose-bisphosphate aldolase and phosphofructokinase which are essential for homolactic acid fermentation. Hence, the heterofermentative metabolism of *L*. *buchneri* CD034 could be confirmed by reconstruction of metabolic pathways involved in lactic acid fermentation [12]. Application of *L*. *buchneri* CD034 as an inoculum for grass silage resulted in changes in the chemical parameters of the silage. Inoculated silage featured higher levels of acetate, lower levels of lactic acid and a slightly higher pH value [13]. Also changes within the microbial ensiling communities were induced, namely lower levels of yeasts and higher levels of LAB were detected in inoculated silage [13]. Moreover, *L*. *buchneri* CD034 was found to become a dominant member within microbial ensiling communities [13].

The change from anaerobic to aerobic conditions after opening of the silo is a crucial event for its quality. It would be interesting to identify genetic systems that enable *L*. *buchneri* CD034 to positively affect the aerobic stability of silage. Metatranscriptomic analyses of silage communities would be the method of choice to address this question. However, it is supposed that harvesting of cells would strongly influence bacterial transcriptomes. Hence, in this study, the influence of oxygen upon gene expression of *L*. *buchneri* CD034 grown under conditions simulating the ensiling process was analyzed by high-throughput transcriptome sequencing. This method provides the advantage that design of a microarray is not required and that a resolution at the nucleotide sequence level and a high dynamic range is achieved. Differential RNA-Seq experiments have recently been applied to analyze the influence of different culture conditions upon gene expression in different microorganisms [14–17]. Using this method, highly transcribed genes can be identified and differentially transcribed genes under aerobic *vs*. anaerobic conditions were investigated. Moreover, oxygen-dependent expression of genes playing a role in lactic acid fermentation was analyzed.

## Material & Methods

### Cultivations of *L*. *buchneri* CD034

Fermentations with *L*. *buchneri* CD034 were performed in continuously stirred tank reactors (CSTR) with controlled gas phase compositions. The Fermenters (2 biological replicates) (DASGIP cultivation system, Eppendorf, Germany) contained 1 l of MRS medium [18] with different carbohydrates (12 g/l glucose, 6 g/l xylose and 1 g/l arabinose) instead of only glucose and an elevated pH of 7.0. Temperature was controlled at 37°C which is the growth temperature recommended by culture collections such as the DSMZ or the ATCC for *L*. *buchneri* strains [19,20]. The fermentation broth was stirred at constant speed of 400 rpm and the gas flow was set to 2.00 ± 0.06 l/h (MX4/4, DASGIP cultivation system, Eppendorf, Germany). The pH, dO_2_ and redox potential were measured but not controlled. The fermenter was inoculated with 20 ml of overnight cultures. Samples were taken after 2.5, 5, 7, 10, 18.5 and 21 hours of fermentation. One sample was taken to determine the optical density (OD) of the broth and for HPLC analysis of the supernatant to determine the concentration of organic acids (lactate, acetate) and carbohydrates (glucose, xylose, arabinose). After 7, 10, 18.5 and 21 hours of fermentation a second sample (3 ml) was taken and mixed with 10 ml of a -40°C methanol/HEPES solution [21] directly after sampling to stop transcription and RNA degradation. The fermentation was first operated under aerobic conditions. Fermenters were aerated with 21% of oxygen (and 79% of nitrogen). After 7 h of fermentation the conditions were changed to an anaerobic environment. Hence, the medium was aerated with 2% carbon dioxide (and 98% of nitrogen). After another 12.5 h, fermenters were again aerated with 21% of oxygen.

### Measurement of metabolite and carbohydrate concentrations by HPLC

The concentrations of carbohydrates (glucose, xylose, arabinose) and organic acids as metabolites (lactate, acetate) in the culture broth were determined by HPLC analysis (Shimadzu, Korneuburg, Austria) with a Rezex ROA-Organic Acid H^+^ column (300 mm x 7.8 mm, Phenomenex, USA) and a refraction index detector (RID-10A, Shimadzu, Korneuburg, Austria). The column was operated at 60°C temperature, 1.0 ml/min flow rate and 0.004 M H_2_SO_4_ as mobile phase. HPLC samples were prepared by adding 100 μl of 0.04 M H_2_SO_4_ to 900 μl culture supernatant. Subsequently the samples were filtrated and 10 μl were injected for analysis. Standard solutions injected for quantification were treated in the same way to ensure comparability. The detection limit for HPLC measurements is given by the lowest amount which was injected of a standard substance (50 mg/l).

### RNA isolation, sequencing library generation and RNA sequencing

Total RNA was isolated from harvested cells. Cells were disrupted by ribolyzation applying Lysing Matrix B tubes (MP Biomedicals). Total RNA was isolated by applying the RNeasy Mini Kit (Qiagen) according to the protocol provided by the manufacturer.

Messenger-RNA (mRNA) was enriched by means of the RiboZero rRNA Removal Kit for Bacteria by applying 3 μg of RNA per sample following the protocol of the manufacturer. Subsequently, RNA was precipitated with ethanol and dissolved in 5 μl of water. For library generation the ‘TruSeq Stranded mRNA Sample Prep Kit’ (Illumina) was applied starting within the ‘Purify and Fragment mRNA step’ of the manufacturer’s protocol. Sequencing libraries were sequenced in two lanes on the Illumina HiSeq 1500 System applying the Rapid Mode together with other samples aiming to yield about 30 x 10^6^ sequences per sample.

### Sequence data processing and data evaluation

Obtained sequences were mapped onto the reference genome sequence of *L*. *buchneri* CD034 [22] by means of BWA [23]. Moreover, sequences were also mapped onto the sequence of the rRNA operon (pos. 1,592,475–1,597,380) to estimate the abundance of sequences originating from 16S and 23S rRNAs. Alignments were processed by means of samtools [24] and then imported into the RNA-Seq analysis platform ReadXplorer [25]. Within this platform RPKM values were calculated. Differential transcription levels of genes as defined by annotated coding sequences (CDS) were also calculated within ReadXplorer by means of DeSeq [26]. For each gene DESeq computes the fold-change which—in this case—represents the change of transcriptional levels between aerobic and anaerobic conditions (a fold change > 1 describes an increasing transcriptional level under aerobic conditions while a fold change < 1 describes a decreasing transcriptional level under aerobic conditions). Additionally a corresponding p-value is computed which can be used for a statistical test by accepting or rejecting the assumption that gas phase composition changes do not influence gene expression.

The data discussed in this publication have been deposited in NCBI's Gene Expression Omnibus [27] and are accessible through GEO Series accession number GSE67802. Raw sequence data are available at the NCBI Sequence Read Archive (Accession No. SRP057107).

### Search for binding sites of regulatory proteins in upstream regions of differentially expressed genes

Sequences of regulatory sites from members of the family *Lactobacillaceae* for PerR and Rex were downloaded from the RegPrecise database [28] and were used for computation of position weight matrices by means of MEME [29]. Resulting models were used to search for those motifs in upstream sequences (CDS start—300 bp) of genes of interest by means of FIMO [29]. Motifs with a p-value below 0.0001 and the right orientation were considered to be putative binding sites of PerR or Rex.

## Results and Discussion

### Controlled cultivation of *Lactobacillus buchneri* CD034 under anaerobic and aerobic conditions

**Fig 1:**
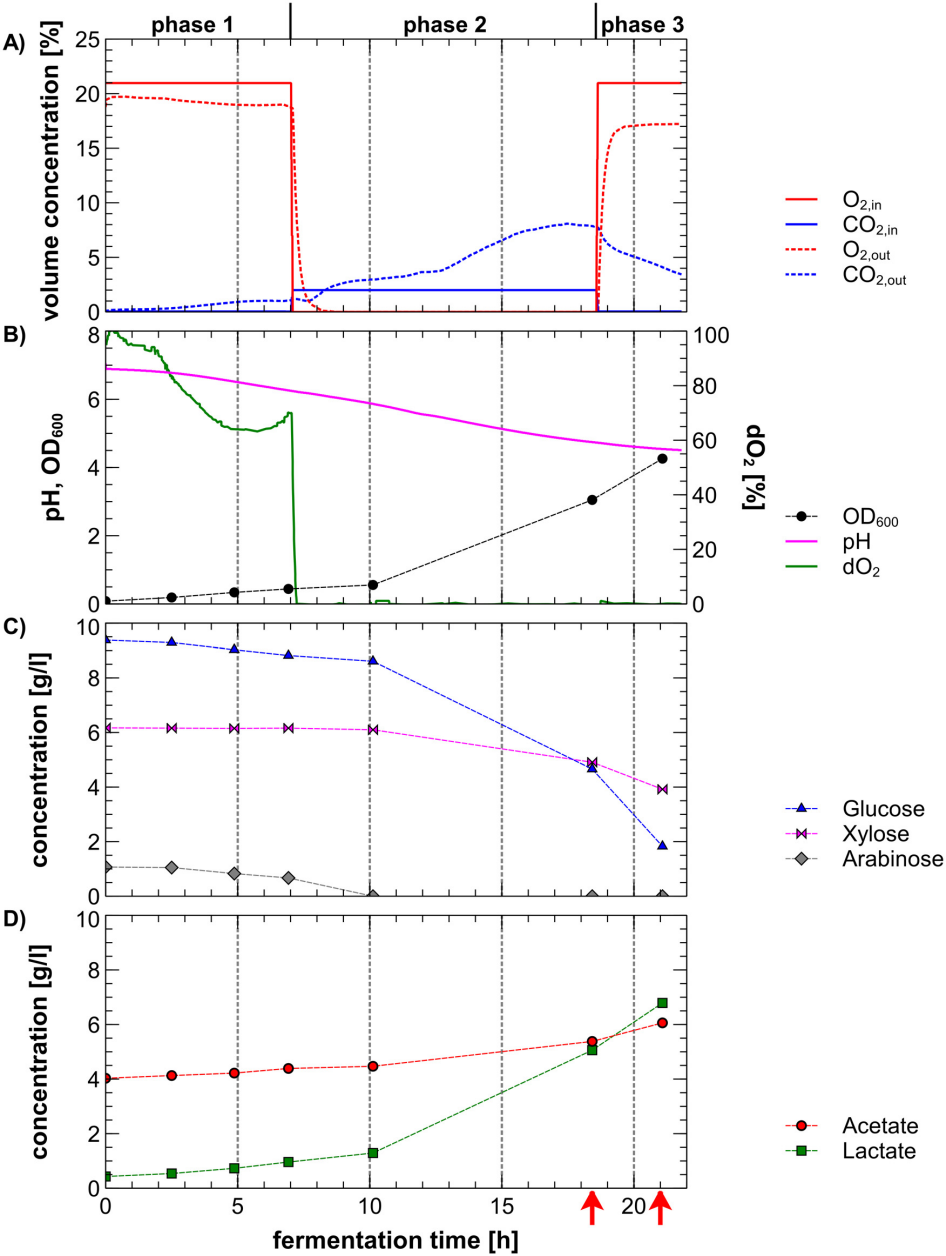
Fermentation of *L*. *buchneri* CD034 grown under aerobic and anaerobic conditions. The fermentation was separated in three phases: phase 1 represents aerobic, phase 2 anaerobic and phase 3 again aerobic conditions with respect to the oxygen concentration of the gas inlet (O_2,in_). (A) Gas phase composition during fermentation as described by oxygen and carbon dioxide concentrations in the gas inlet (O_2,in_, CO_2,in_) and in the gas outlet (O_2,out_, CO_2,out_). (B) pH, dissolved oxygen concentration in the medium (dO_2_) and optical density (OD). (C) Concentration of carbohydrates glucose, xylose and arabinose and (D) concentration of organic acids lactate and acetate. Fermentations were performed in duplicates. Arrows indicate sampling time points for RNA-Seq. Parameters shown originate from fermenter 1. For fermenter 2 see S1 Fig.

To analyze the *L*. *buchneri* CD034 transcriptome under aerobic and anaerobic conditions, the strain was cultivated with and without oxygen supply. The fermentation experiment was designed to simulate opening of the silo (exposure to oxygen) after ensiling (anaerobic conditions) to evaluate the transcriptional response of *L*. *buchneri* CD034 under these conditions. Cultivation of *L*. *buchneri* CD034 was done in two independent continuously stirred tank reactors (replicates) with controlled gas phase compositions. Cells were grown in MRS medium with arabinose, xylose and glucose since these sugars are the main sugars present in grass [30]. During fermentation, gas phase composition, the pH-value and the dissolved oxygen content (dO_2_) were measured online while the optical cell density (OD) and concentrations of carbohydrates and organic acids (metabolites) were determined in samples taken at different time points for both fermentations (as indicated in Fig 1 / S1 Fig). Measured values are presented in Fig 1 for fermenter 1 and in S1 Fig for fermenter 2. Gas phase compositions are shown in Fig 1A and parameters reflecting bacterial growth (pH, dO_2_, OD_600_) are shown in Fig 1B. Consumption of carbohydrates and production of lactic acid and acetic acid are displayed in Fig 1C and 1D, respectively.

The fermentations proceeded in three distinct cultivation phases simulating gas phase compositions during the ensiling process (Fig 1A). In the first (aerobic) phase, the system was aerated with 21% of oxygen. Aeration of the system with oxygen concentrations similar to those in air was considered to represent aerobic conditions. After 12.5 h of fermentation, the second phase was initiated which was defined by changing the gas composition of the inlet air to 2% of carbon dioxide (CO_2_) to create anaerobic conditions. This stage stands for creation of anaerobic conditions during the ensiling process by compressing the green fodder and by successive consumption of remaining oxygen and production of carbon dioxide. The second fermentation phase persisted for 11.5 h. After the ensiling process, the silage can be opened and oxygen again has access to the fermented green fodder. Hence, aerobic conditions were again created in the third phase of the fermentation by aeration with 21% of oxygen (O_2_) for another 3 hours. In the gas outlet, also oxygen and carbon dioxide were measured during the fermentation (Fig 1A). Oxygen concentrations in the outlet air were found to feature lower levels than the oxygen concentration of the inlet air during the aerobic fermentation phases. During the first fermentation phase, production of slight amounts of carbon dioxide was observed and increased during the anaerobic fermentation phase. When the system was again aerated with 21% of oxygen, the production of carbon dioxide decreased again.

The pH values as well as the optical density values indicate bacterial growth during all three fermentation phases. During the first aerobic phase bacterial growth could be observed since OD values slightly increased from 0.08 to 0.4. In the second fermentation phase (anaerobic phase), the growth rate of *L*. *buchneri* CD034 further increased, resulting in an OD of 3.1. Aeration did not negatively influence growth since the OD further increased to 4.3 (Fig 1B). The pH dropped quite constantly from pH 7 at the beginning of the fermentation to pH 4.5 at the end of the fermentation. The dissolved oxygen within the fermentation broth decreased during the first aerobic fermentation phase probably due to the beginning of bacterial growth. When anaerobic conditions were created, the dO_2_ stayed at 0% due to the absence of oxygen in the inlet gas (Fig 1B and 1A). During the third fermentation phase, the system again was aerated with 21% of O_2_ (aerobic conditions) (Fig 1A). However, an increasing dO_2_ could not be observed even though oxygen was introduced into the system (Fig 1B). Redox potential measurements also verified the gas phase composition changes. At the end of the anaerobic phase the redox potential had dropped to about -220 mV and then increased to -30 mV during the aerobic phase. Hence, it was concluded that O_2_ was consumed and converted by *L*. *buchneri* CD034 and thus remained within the system since the O_2_ concentration in the gas outlet did not reach the inlet concentration (Fig 1A).

The carbohydrates arabinose, glucose and xylose were consumed during all fermentation phases (Fig 1C). Simultaneous consumption of carbohydrates is also described for other lactobacilli such as *L*. *brevis* [31]. Arabinose was already consumed after 10 hours at the beginning of the anaerobic fermentation phase. Glucose was then preferably metabolized compared to xylose. However, glucose and xylose were not completely depleted during the fermentations (Fig 1C). Hence, effects caused by nutrient deficiency could be excluded.

Metabolism of carbohydrates by heterolactic acid fermentation led to increased concentrations of lactic acid and acetic acid (also a component of the MRS medium) (Fig 1D) causing a decline in the pH-value (Fig 1B). Production of both acids was found to be lowest during the first aerobic phase and increased during the following anaerobic and aerobic phase as cell numbers increased.

Overall, both fermentations (replicates) showed similar developments (Fig 1 / S1 Fig) *L*. *buchneri* CD034 was able to grow under aerobic as well as anaerobic growth conditions and produced lactic acid and acetic acid under these conditions. Inlet gas phase composition changes did not negatively influence growth of the *L*. *buchneri* CD034 culture. However, CO_2_ production seemed to be influenced by the inlet gas phase composition. Consumption of oxygen and simultaneous production of carbon dioxide may allow *L*. *buchneri* CD034 to establish microaerophilic conditions within aerated silage and thereby growth inhibition of aerobic microorganisms is enhanced.

Aeration of the cultures with 21% oxygen showed the uptake of oxygen by *L*. *buchneri* CD034 cells. Therefore, the shift from anaerobic to aerobic conditions was chosen for sampling of the culture since it reflects opening of the silo and creation of aerobic conditions. Samples for the analysis of transcriptional profiles under anaerobic *vs*. aerobic conditions were taken at the end of the anaerobic fermentation phase before oxygen aeration and 2.5 hours after oxygen exposure as indicated in Fig 1 and S1 Fig. This second time point was chosen to ensure complete adaptation of *L*. *buchneri* CD034 cells to aerobic conditions and corresponding tuning of its metabolism.

### Sequencing and analysis of transcriptomes of *L*. *buchneri* CD034 grown under anaerobic and aerobic conditions

To analyze the *L*. *buchneri* CD034 transcriptome under anaerobic and aerobic conditions, samples from the fermentations were taken at the end of the anaerobic phase and 2.5 hours after the onset of aerobic conditions (see Fig 1 and S1 Fig). After sampling (2 fermenters representing biological replicates as indicated in Fig 1 and S1 Fig), total RNA was prepared from harvested cells, mRNA was enriched and then used to construct cDNA sequencing libraries which were sequenced on the Illumina HiSeq system. Enrichment of mRNA was done since commonly a large proportion of bacterial RNA represents ribosomal RNA which is unwanted in studies focusing on differentially transcribed protein-encoding genes.

Sequencing libraries each yielded on average approx. 3.3 x 10^7^ reads of which in general more than 98% could be mapped onto the *L*. *buchneri* CD034 reference genome sequence by means of the BWA (Burrows-Wheeler Aligner) software package (see Table 1). Among these mapped sequences, only 2 to 10% originated from 16S or 23S rRNA genes. Even though rates of rRNA depletion differed slightly, a sufficient depletion of these RNA species in the course of library preparation was confirmed (see Table 1).

**Table 1:** Overview on the sequencing of transcripts (mRNA) isolated from *L*. *buchneri* CD034 grown in two replicate fermenter systems under anaerobic *vs*. aerobic conditions.

			*L*. *buchneri* CD034 genome		*L*. *buchneri* CD034 *rrn* operon	
Sampling time	Fermenter	Sequencing reads total	Sequencing reads mapped	Percentage [%]	Sequencing reads mapped	Percentage of mapped reads [%]
18.5 h (anaerobic)	1	33,134,361	32,521,030	98.15	740,994	2.28
	2	29,873,616	29,343,326	98.22	3,131,915	10.67
21 h (aerobic)	1	38,470,216	37,769,788	98.18	971,578	2.57
	2	31,368,998	30,819,068	98.25	598,180	1.94

Sequence mappings were further evaluated within the ReadXplorer platform [25]. First, the number of reads that could be mapped onto protein-encoding genes was calculated (see S1 Table). Based on the read count data, RPKM (reads per kilobase of transcript per million mapped reads) values for these genes were calculated, which allows normalization for gene lengths and sequence library sizes [32] (see S1 Table). In general, *L*. *buchneri* CD034 genes feature RPKM values in a range from 0.25 to approx. 62,000. Approximately, 50% of the genes transcribed under anaerobic conditions feature RPKM values below 154, while 50% of the genes transcribed upon oxygen exposure feature RPKM values below 163. Hence, a large proportion of genes is transcribed at low levels. With the exception of LBUCD034_2165, the 25 most frequently transcribed protein-encoding genes featured similar RPKM values under both growth conditions (Table 2) and are encoded on the chromosome or the large plasmid pCD034-3. The gene featuring the highest level of transcription encodes the SlpB S-layer protein. This surface layer protein of *L*. *buchneri* CD034 has been described as a unique protein compared to *L*. *buchneri* NRRL-B-30929 [12] and was probably introduced into the *L*. *buchneri* CD034 genome by a transduction event. S-layer proteins enable adhesion of cells, stabilize the membrane and form a barrier against bacteriophages or chemicals [33]. The DNA binding protein HU was found to feature the second highest transcriptional level. It is a histone-like protein and interacts with DNA during processes such as replication, supercoiling or repair [34]. Transcriptional levels of both genes indicate their importance for growth of *L*. *buchneri* CD034. Additionally, further gene products encoded by highly transcribed chromosomal genes (Table 2) play a role in processes essential for basic cell functions including protein biosynthesis (ribosomal proteins, elongation factors), energy metabolism (ATP synthase subunit), lactic acid fermentation and pyruvate or lactate metabolism (glyceraldehyde-3-phosphate dehydrogenase, enolase, pyruvate dehydrogenase complex components, D-lactate dehydrogenase) or concern cell wall composition (S-layer proteins, LrgAB proteins [35]). Moreover, the two genes LBUCD034_2165 and LBUCD034_1844 were identified to be among the most frequently transcribed genes. They were annotated to encode hypothetical proteins. Both CDS feature a ribosomal binding site upstream of their start-codon and did not share any similarity to entries of the RFAM database collecting RNA families [36]. Hence, it can be concluded that these genes most probably encode proteins. Both proteins are rather small (58 and 73 amino acids), do not contain conserved domains and homologous proteins were only identified in some other *Lactobacillus* species. Even though the function of these short gene products still is unknown, they seem to be of importance for at least some lactobacilli.

**Table 2:** The 25 most abundantly transcribed genes of *L*. *buchneri* CD034 cultivated under aerobic *vs*. anaerobic conditions.

	Locus tag (Gene)	Product	RPKM^1^ anaerobic	RPKM^1^ aerobic
1	LBUCD034_1608 (*slpB*)	Surface layer protein	59,082.00	62,393.44
2	LBUCD034_1179	DNA-binding protein HU	38,783.96	50,281.67
3	LBUCD034_1448 (*gapA1*)	Glyceraldehyde 3-P dehydrogenase	17,388.62	16,673.23
4	LBUCD034_2143	Cold shock-like protein	15,725.65	12,988.81
5	LBUCD034_p0047	Hypothetical protein	15,693.50	17,154.48
6	LBUCD034_p0049	D-arabitol-P dehydrogenase	15,268.25	17,537.38
7	LBUCD034_2165	Hypothetical protein	15,152.15	45,297.97
8	LBUCD034_0801 (*tuf*)	Elongation factor Tu	13,947.76	13,734.86
9	LBUCD034_p0048	Fructose permease	12,978.79	14,111.96
10	LBUCD034_1485	Putative effector of murein hydrolase LrgA	10,596.33	12,131.98
11	LBUCD034_1146 (*rpsU*)	30S ribosomal protein S21	10,576.47	11,468.02
12	LBUCD034_1262 (*rpmB*)	50S ribosomal protein L28	10,170.23	9,780.19
13	LBUCD034_0782 (*pdhB*)	Pyruvate dehydrogenase E1 component subunit β	10,161.31	9,201.17
14	LBUCD034_0781 (*pdhA*)	Pyruvate dehydrogenase E1 component subunit α	10,056.08	9,147.41
15	LBUCD034_0797 (*rpsT*)	30S ribosomal protein S20	9,923.50	9,740.80
16	LBUCD034_2468 (*rpmH*)	50S ribosomal protein L34P	9,703.66	9,554.52
17	LBUCD034_1486	Putative effector of murein hydrolase LrgB	9,663.10	11,249.36
18	LBUCD034_0784 (*pdhD*)	Dihydrolipoamide dehydrogenase	9,597.14	8,803.51
19	LBUCD034_0783 (*pdhC*)	Pyruvate dehydrogenase E2 component	9,043.26	8,141.00
20	LBUCD034_p0050	Fructokinase	8,626.95	10,419.90
21	LBUCD034_0163	Hypothetical protein	8,621.40	7,104.43
22	LBUCD034_1641	50S ribosomal protein L31 type B	7,588.84	6,690.24
23	LBUCD034_1104 (*ldhD2*)	D-lactate dehydrogenase	7,309.54	6,517.15
24	LBUCD034_p0046 (*pgi*)	Glucose 6-P isomerase	7,174.93	7,865.74
25	LBUCD034_0999 (*atpC*)	F0F1-type ATP synthase, ε subunit	7,050.11	5,440.49

^1^ Reads per kilobase of transcript per million mapped reads.

Some genes encoded on the large plasmid pCD034-3 were found to be among the most abundantly transcribed genes. Corresponding gene products were predicted to be involved in glucose conversion (fructokinase), lactic acid fermentation (glucose-6-phosphate isomerase) and D-arabitol phosphate conversion (D-arabitol-phosphate dehydrogenase). D-arabitol usually is a product of xylose metabolism. Hence, this plasmid may supplement metabolic pathways and reactions for reduction equivalent regeneration already encoded on the *L*. *buchneri* CD034 chromosome.

**Fig 2:**
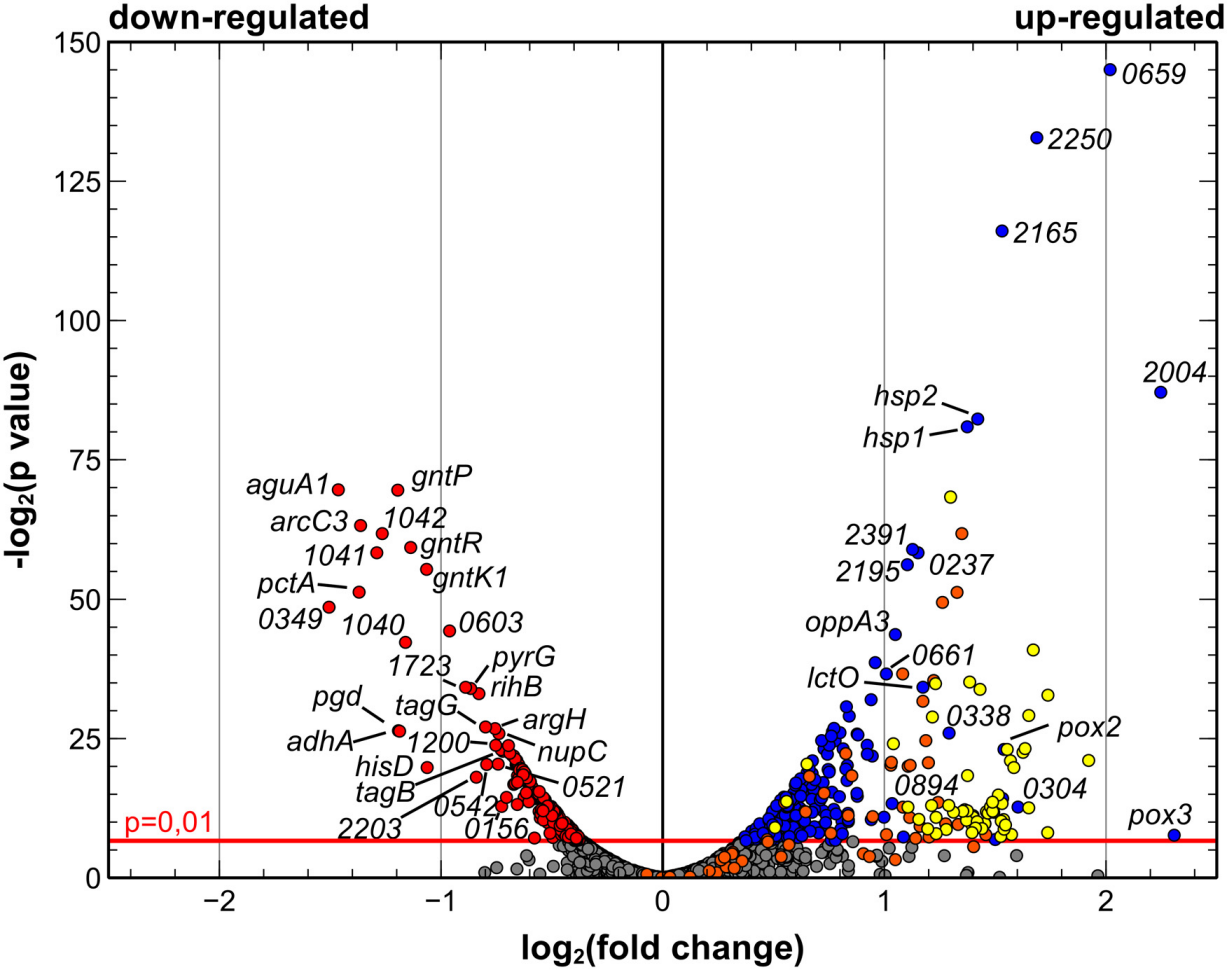
Volcano plot representing transcriptional levels for *L*. *buchneri* CD034 genes under aerobic *vs*. anaerobic conditions. For each protein encoding gene the −log_2_(p-value) is plotted against its log_2_(fold change). Genes up-regulated (p < 0.01) under aerobic conditions are colored blue while down-regulated genes are colored red. Genes found to be located between bacteriophagous attachment sites are colored in yellow (*ΦLbu-1*) and orange (*ΦLbu-2*) irrespective of their relative transcriptional levels. For genes with gene names that were found to be highly up- (see Table 3) or down- (see Table 4) regulated the gene name is given. Abbreviations represent gene names: *pox2*, *-3*: pyruvate oxidase; *lctO*: lactate oxidase; *hsp1*, *-2*: molecular chaperones; *oppA3*: oligopeptide transport system, substrate binding protein; *arcC3*: carbamate kinase; *aguA1*: agmatine deiminase; *pctA*: putrescine carbamoyltransferase; *gntP*: H+/gluconate symporter; *gntR*: gluconate operon transcriptional regulator; *gntK1*: gluconokinase; *pyrG*: CTP synthetase; *rihB*: ribosylpyrimidine nucleosidase; *pgd*: 6-phosphogluconate dehydrogenase; *adhA*: alcohol dehydrogenase; *hisD*: histidinol dehydrogenase; *tagG*: teichoic acid permease; *tagB*, teichoic acid biosynthesis protein; *argH*: argininosuccinate lyase; *nupC*: pyrimidine specific nucleoside symporter. For genes lacking gene names the index numbers of their respective locus tags (LBUCD034_XXXX) are given.

**Fig 3:**
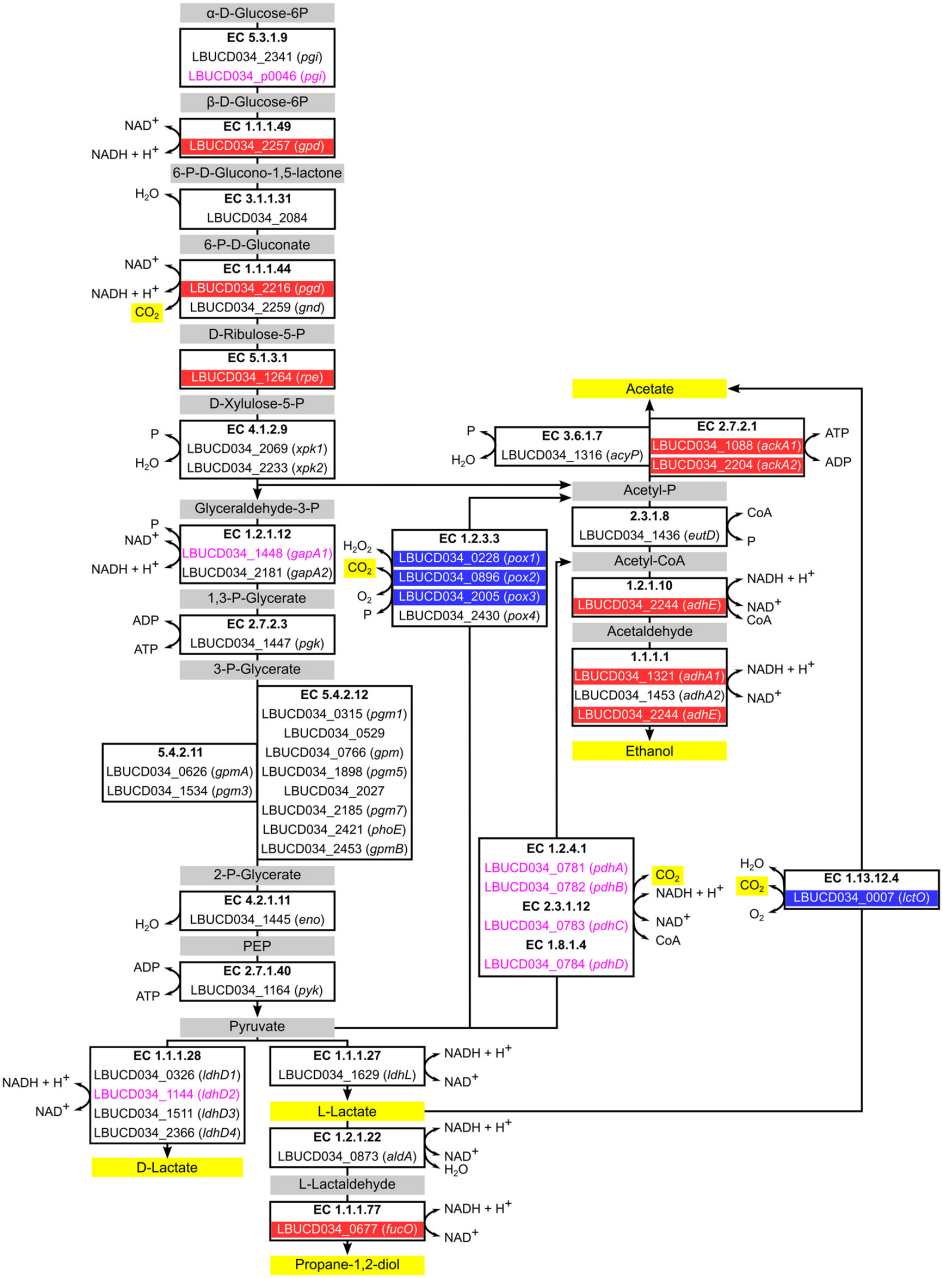
Metabolic pathway chart of gene products encoded by *L*. *buchneri* CD034 and metabolites that play a role in heterolactic fermentation. Genes are represented by their locus tag and the gene name, if applicable. Locus tags and gene names with highlighted background indicate genes that were found to be up-regulated (blue) or down-regulated (red) under aerobic *vs*. anaerobic conditions. Locus tags and gene names printed in pink indicate genes that were found to be highly transcribed. Pathway end products (yellow) and intermediates (gray) are also highlighted.

To identify *L*. *buchneri* CD034 genes that featured a differential transcriptional level in response to aerobic conditions compared to anaerobic conditions, the DESeq module [26] within the ReadXplorer platform was applied (see S1 Table for computed fold-changes and p-values). Results are displayed as volcano plot in Fig 2. In total, 283 genes were significantly (p < 0.01) up-regulated while 198 were down-regulated after exposure to oxygen. A large number of up-regulated transcripts under aerobic conditions can be attributed to genes of two prophages (Φ *Lbu-1 and* Φ *Lbu-2*) that had integrated into the *L*. *buchneri* CD034 genome [12] and which are marked in yellow or orange in Fig 3. The high number of up-regulated phage genes may be interpreted as a stress response upon oxygen exposure. In *Staphylococcus aureus*, also a member of the class *Bacilli*, it has been shown that transcription of bacteriophage genes is part of a general SOS response induced by stress factors [37]. Bacteriophage genes were not further considered in the context of differential gene transcription under aerobic *vs*. anaerobic conditions.

### *Lactobacillus buchneri* CD034 genes up-regulated under aerobic *vs*. anaerobic growth conditions

Genes found to be up-regulated under aerobic conditions compared to anaerobic conditions were further evaluated to investigate the influence of oxygen onto transcription in *L*. *buchneri* CD034. Twenty-one of the most up-regulated, non bacteriophage genes (fold change > 2) upon aerobic conditions are functionally annotated as genes encoding oxidases, chaperones, hypothetical proteins or enzymes featuring other predicted activities (see Table 3). RPKM values for these genes under anaerobic and aerobic conditions are also given in Table 3. The transcriptional level of some of these genes is very low (LBUCD034_0338), whereas others are highly transcribed (LBUCD034_2165).

**Table 3:** Genes of *L*. *buchneri* CD034 found to be highly^1^ up-regulated under aerobic *vs*. anaerobic conditions.

Gene^2^	Function	Fold change	RPKM^3^ anaerobic	RPKM^3^ aerobic
**(Per) oxidases**				
LBUCD034_2005 (*pox3*)	Pyruvate oxidase (1.2.3.3)	5.0	39.53	203.04
LBUCD034_0896 (*pox2*)	Pyruvate oxidase (1.2.3.3)	2.9	60.71	182.68
LBUCD034_0007 (*lctO*)	L-lactate oxidase (1.13.12.4)	2.3	15.28	35.66
**Chaperones**				
LBUCD034_0267 (*hsp1*)	Molecular chaperone	2.7	167.55	463.93
LBUCD034_0266 (*hsp2*)	Molecular chaperone	2.6	224.95	602.96
**Others**				
LBUCD034_0338	putative hydrolase	2.5	8.96	22.68
LBUCD034_0237	Isochorismatase	2.2	204.95	470.96
LBUCD034_1974 (*xylE*)	D-xylose-proton symporter	2.1	177.45	390.25
LBUCD034_2391	3-hydroxybutyryl-CoA dehydrogenase	2.2	286.13	646.25
LBUCD034_2329 (*oppa3*)	Oligopeptide transport system, substrate binding protein	2.1	42.65	91.20
LBUCD034_0661	Short chain dehydrogenase	2.0	69.46	144.65
**(conserved) hypothetical proteins**				
LBUCD034_2004	-	4.7	95.11	467.37
LBUCD034_0659	-	4.1	132.64	555.74
LBUCD034_2250	-	3.2	950.58	3164.16
LBUCD034_0303	-	2.9	187.87	563.16
LBUCD034_0304	-	3.0	234.26	737.07
LBUCD034_0305	-	2.8	265.63	778.67
LBUCD034_2165	-	2.9	15152.75	45297.97
LBUCD034_2195	-	2.1	422.88	939.43
LBUCD034_1600	-	2.1	25.70	56.41
LBUCD034_0894	-	2.0	68.84	145.64

^1^ fold change > 2

^2^ genes related to bacteriophages are not shown.

^3^ Reads per kilobase of transcript per million mapped reads.

Several genes encoding oxidases (pyruvate oxidases, lactate oxidase) were transcriptionally up-regulated under aerobic conditions. Corresponding gene products are supposed to fix oxygen in carbon dioxide, water and hydrogen peroxide while producing acetyl-phosphate or acetate (Fig 3). Hence, presence of oxygen most probably leads to an increased production of acetate in *L*. *buchneri* CD034 as described for other *Lactobacillus* species [38,39,2,3].

Even though *L*. *buchneri* CD034 encodes enzymatic reactions to respond to the presence of oxygen, oxygen also seems to provoke some general stress responses as genes encoding chaperones (*hsp1*, *hsp2*) were found to be up-regulated under these conditions.

Oxidation of pyruvate to acetyl-P by pyruvate oxidases (*pox2*, *pox3*) leads to the production of hydrogen peroxide (H_2_O_2_) featuring antimicrobial properties. Hydrogen peroxide also is a precursor of reactive oxygen species (ROS) such as organic hydroperoxides. Even though lactobacilli are described to produce H_2_O_2_ under aerobic as well as under anaerobic conditions and feature a natural resistance against this molecule, they need mechanisms to get rid of it [40–42]. Hence, genes found to be up-regulated (fold change > 1, p-value < 0.01, S1 Table), and having a functional annotation were additionally screened for potential functions in the context of oxygen conversion and ROS decomposition. Genes identified were predicted to play a role in decomposition of hydrogen peroxide (*npr*) or organic hydroperoxides (*ohrA*, *ohrR*, *LBUCD034_2220*), oxygen consumption (*cydA*, *cydB*, *pox1*, *LBUCD034_0437*, *LBUCD034_0888*, *LBUCD034_0494*), glutathione synthesis and reduction (*gshR1*, *gshR3*, *gshA1*, *gshA2*, *gshA3*, *LBUCD034_0719*) or in ROS protection by encoding proteins like a non-heme iron containing ferritin-like protein (LBUCD034_0195), a manganese transport protein (LBUCD034_0386) or a universal stress protein (LBUCD034_1367). Upstream sequences of genes discussed above were also analyzed for the presence of binding sites for the regulatory proteins PerR (LBUCD034_2086) and Rex which are known to be involved in transcriptional regulation of genes in response to oxygen availability in lactobacilli [43]. However, only the NADH peroxidase gene (*npr*) was found to possess a putative PerR binding site overlapping with its putative -10 promoter motif. The *perR* gene itself (LBUCD034_2086) also features a corresponding PerR-binding motif in its non-transcribed upstream region and was also found to be up-regulated upon oxygen exposure (fold change 1.58, see S1 Table). Hence, it is very likely that transcription of the *npr* gene is regulated by the hydrogen peroxide sensitive LBUCD034_2086 gene product (PerR) which also regulates its own transcription.

Pyruvate oxidase genes are also described to be up-regulated under stationary growth for other lactobacilli [2]. To exclude growth phase change influences, results of this study were compared to proteomic or microarray studies analyzing gene expression in the stationary phase of *L*. *plantarum* [44], *L*. *casei* [45], *L*. *acidophilus* [46] and *L*. *rhamnosus* [47]. Expression changes of genes described in at least two of these studies were compared to those of this study. The majority of these genes (e.g. *htrA*, *luxS*, *grpE*, *dnaK*, *clpP1*, *groEL*, *ldhL*, *ptsH2*) was found not to be differentially expressed in *L*. *buchneri* CD034 in this study. Hence, transcriptional changes influenced by growth phase changes can be excluded.

Even though *L*. *buchneri* CD034 is a fermentative organism, it still can utilize oxygen by conversion of compounds such as pyruvate and lactate. This reduces the amount of oxygen and hence lowers its deleterious potential. However, enzymatic reactions also produce ROS such as hydrogen peroxide. Hydrogen peroxide and/or organic peroxides such as peracetic acid feature strong antimicrobial properties and may also be a factor contributing to the high aerobic stability of silage inoculated with *L*. *buchneri*. Typical enzymes described for hydrogen peroxide decomposition in lactobacilli (catalases or pseudocatalases [48,49]) were not identified in the *L*. *buchneri* CD034 genome. However, *L*. *buchneri* CD034 encodes several other genes for protection against ROS, e.g. *via* glutathione metabolism or decomposition of peroxides and additional protective proteins which were also found to be up-regulated under aerobic conditions.

### *Lactobacillus buchneri* CD034 genes down-regulated under aerobic *vs*. anaerobic growth conditions

Genes that were down-regulated under aerobic conditions generally show a lower fold-change value than up-regulated genes. The 20 most down-regulated genes (transcriptional expression fold-change values < 0.6) are shown in Table 4. Encoded gene products are involved in a variety of different metabolic pathways. Different genes play a role in amino acid metabolism, e. g. they encode amino acid transporters or enzymes playing a role in agmatine metabolism. Three of the down-regulated genes encode dehydrogenases that are involved in NADH regeneration. Gene products of the remaining genes are supposed to play a role in the gluconate, pyrimidine and cell wall component metabolism.

**Table 4:** Genes of *L*. *buchneri* CD034 found to be highly^1^ down-regulated under aerobic *vs*. anaerobic conditions.

Gene	Function	Fold change	RPKM^2^ anaerobic	RPKM^2^ aerobic
**Arginine metabolism / Agmatine conversion to NH**_**3**_ **, pH homeostasis**				
LBUCD034_0349	Putrescine / Agmatine symporter	0.35	95.42	34.82
LBUCD034_0350 (*aguA1*)	Agmatine deiminase (3.5.3.12)	0.36	103.49	38.87
LBUCD034_0351 (*arcC3*)	Carbamate kinase (2.7.2.2)	0.38	105.56	42.50
LBUCD034_0348 (*pctA*)	Putrescine carbamoyltransferase	0.39	128.56	51.54
LBUCD034_0807 (*argH*)	Argininosuccinate lyase	0.59	689.90	422.10
**Amino acid transport**				
LBUCD034_1041	ABC type amino acid transporter, ATPase component	0.41	142.32	60.12
LBUCD034_1042	ABC type amino acid transporter, permease component	0.42	161.37	69.38
LBUCD034_1040	ABC type amino acid transporter, permease component	0.41	98.24	45.39
**Dehydrogenases**				
LBUCD034_1321 (*adhA*)	Alcohol dehydrogenase	0.44	6166.62	2782.13
LBUCD034_0669 (*hisD*)	Histidinol dehydrogenase	0.48	15.40	7.62
**Gluconate metabolism**				
LBUCD034_2216 (*pgd*)	6-phosphogluconate dehydrogenase	0.44	4130.19	1870.68
LBUCD034_1726 (*gntP*)	H+/gluconate symporter	0.44	1574.29	710.27
LBUCD034_1724 (*gntR*)	Gluconate operon transcriptional regulator	0.45	440.02	206.56
LBUCD034_1725 (*gntK1*)	Gluconokinase	0.48	1230.04	607.47
**Pyrimidine metabolism**				
LBUCD034_0603	Xanthine / uracil / vitamin C permease	0.51	1376.35	730.64
LBUCD034_1645 (*pyrG*)	CTP synthetase	0.55	545.29	309.58
LBUCD034_0604 (*rihB*)	Ribosylpyrimidine nucleosidase	0.56	1933.60	1124.73
LBUCD034_0602 (*nupC*)	Pyrimidine specific nucleoside symporter	0.60	2938.77	1820.83
**Cell wall component synthesis**				
LBUCD034_1141 (*tagB*)	Teichoic acid biosynthesis protein	0.60	270.79	168.99
LBUCD034_1142 (*tagG*)	Teichoic acid permease	0.57	211.64	356.07
LBUCD034_1723	Lysozyme M1	0.54	315.16	175.59
**Others**				
LBUCD034_1200	Isopentyl pyrophosphate isomerase	0.59	216.56	132.83
LBUCD034_0542	Na+/H+ antiporter nhaC	0.57	49.53	29.54
LBUCD034_0521	Hydrolase	0.60	467.46	288.31
LBUCD034_0156	Hypothetical protein	0.60	4443.37	2772.63
LBUCD034_2203	Predicted membrane protease	0.56	46.75	26.97

^1^ fold change ≤ 0.6

^2^ Reads per kilobase of transcript per million mapped reads.

Genes most obviously down-regulated upon oxygen exposure were predicted to be involved in the conversion of agmatine to ammonia, putrescine and carbon dioxide (Table 4). This pathway has been described for closely related lactic acid bacteria to be responsible for cytoplasmic pH homeostasis [50–52]. As a result of agmatine degradation, two molecules of ammonia and one molecule of ATP are generated [50]. Ammonia molecules can further react with free protons (H^+^) to generate ammonium and accordingly alkalinization of the cytoplasm occurs. Agmatine cannot be synthesized by *L*. *buchneri* CD034 because the required arginine decarboxylase is not encoded in its genome. Hence, it remains unclear whether there are side reactions which are catalyzed by these enzymes in agmatine-free MRS medium. Simultaneously, genes playing a role in generation of arginine from aspartate and citruline (*argGH*) (see Table 4, S1 Table) were found to be down-regulated under aerobic conditions. This means that there might be an increased production of arginine under anaerobic conditions. Arginine in turn can be metabolized *via* the arginine deiminase pathway which also leads to the formation of ammonia. Lactic acid bacteria may also use this pathway to maintain pH homeostasis [48].

Two other mechanisms which might also influence the intracellular pH were identified to be down-regulated upon oxygen exposure at the transcriptional level: an H^+^/Na^+^ ion antiporter and an H^+^/gluconate symporter (Table 4). The gluconate symporter was down-regulated together with genes involved in transformation of gluconate to D-ribulose-5-phosphate, a precursor for final steps in lactic acid fermentation. A similar gene cluster was found in *Haemophilus influenzae* featuring higher transcriptional levels at high pH values [53]. It cannot be excluded that these genes might have further functions besides gluconate utilization and pH homeostasis [53].

Among the most down-regulated genes, three genes were annotated to encode dehydrogenases (*adhA*, *hisD*, *pgd*). These play a role in redox reactions involving an alcohol compound, histidinol or 6-phospho-D-gluconate as substrates. These reactions enable oxidation of NADH and lead to the reduction of substrate molecules. Reduction of substrate molecules allows the acceptance of two electrons and binding of free protons.

### *L*. *buchneri* CD034 genes involved in lactic acid formation and their change in transcriptional activity under aerobic *vs*. anaerobic growth conditions

Lactic acid formation is the essential pathway for conservation of green fodder during the anaerobic ensiling process and enzymes predicted to be involved in lactic acid fermentation are encoded in *L*. *buchneri* CD034 [12,54]. Corresponding genes were inspected regarding their transcriptional response upon oxygen exposure (Fig 3). Some of these genes *(gapA1*, *pdhABCD*, *pgi*, *ldhD2*, *LBUCD034_p0050*) were found to be highly transcribed irrespective of the oxygen status of the cell (see section 4.2) indicating the importance of these metabolic pathways. Results of the differential transcriptome analysis were evaluated to determine whether transcription of corresponding genes was influenced by oxygen availability in *L*. *buchneri* CD034 (fold change ≠ 1, p-value < 0.01, S1 Table). For the conversion of α-D-glucose-6-phosphate to L- or D-lactate, 13 enzymatic reactions are required. Corresponding enzymes are encoded by 31 genes which is due to the presence of enzyme isoforms for certain reactions. Only three of these genes were found to be differentially down-regulated upon oxygen exposure. These encode two dehydrogenases and one epimerase, while an isoform of the phosphoketolase is encoded by a gene which transcription is independent of the oxygen availability. Also genes encoding enzymes of the pyruvate dehydrogenase complex were not found to be differentially expressed.

However, oxygen presence caused increased transcriptional levels of genes playing a role in conversions of pyruvate or lactate to acetyl phosphate or acetate. Acetyl phosphate can be further metabolized to acetate or ethanol. The *acyP* gene encoding an acetyl-phosphatase was not found to be differentially transcribed under the conditions tested while the acetate kinase genes *ackA1* and *-2* were down-regulated upon exposure of *L*. *buchneri* CD034 to oxygen. Transcription of genes involved in acetyl-phosphate to ethanol conversion was not dependent on oxygen availability (*eutD*, *adhA2*) or was down-regulated (*adhE*, *adhA1*) under aerobic conditions. However, the genes *ackA1*, *-2*, *adhE* and *adhA* were still found to be transcribed under aerobic conditions (see S1 Table) and ongoing acetyl-phosphate metabolism catalyzed by the encoded enzymes is likely to occur. The *adhE* gene, which encodes a bifunctional acetaldehyde-CoA/alcohol dehydrogenase, was identified to possess a Rex binding site in its upstream region. The corresponding transcriptional repressor Rex was described to sense NADH/NAD^+^ levels and to be involved in regulation of anaerobic metabolism in a number of bacteria [55]. Overall, only a small number of oxygen dependent genes regulated by known regulators PerR and Rex were identified.

Differential analysis of transcriptional levels of *L*. *buchneri* CD034 genes under anaerobic and aerobic conditions showed that the majority of genes playing a role in formation of lactic acid are not affected by oxygen. Metabolic pathways involved in lactic acid fermentation are required under anaerobic as well as aerobic conditions by lactobacilli. Corresponding pathways are of importance for i) utilization of carbon sources, ii) generation of energy and iii) maintenance of the redox potential of the cell [56].

Aerobic stability of silage inoculated with *L*. *buchneri* has been attributed to the production of acetate, which features antimicrobial properties [11,10]. Acetate is not only a product of hetero-fermentative lactic acid metabolism but also of aerobic oxidation of lactate and pyruvate. *L*. *buchneri* CD034 genes encoding these enzymes were found to be up-regulated upon oxygen exposure which could lead to increased formation of acetic acid after opening of the silo. Lactic acid can serve as a carbon source for spoiling microorganisms such as yeast or fungi. Hence, oxidation of lactic acid by *L*. *buchneri* and related species would reduce the concentration of this metabolite in silage that has been opened.

## Conclusion

Transcriptome analyses showed that *L*. *buchneri* CD034 responded to the availability of oxygen, presumably to prevent deleterious effects caused by oxygen exposition. *L*. *buchneri* CD034 encodes several genes predicted to be involved in coping with aerobic conditions and low pH stress. Corresponding features presumably are crucial for its competitiveness within microbial ensiling communities [13]. However, importance of agmatine during the ensiling process and hydrogen peroxide or peroxy acids in silages after oxygen exposition has not been surveyed and represents a future research topic to gain further insights into factors specifying performance of inoculant strains in ensiling processes. For a small number of oxygen dependent genes, corresponding regulator proteins were suggested. However, for a larger number of genes, it is still unclear how they are regulated in response to oxygen. Regulator fishing or systematic mutational analyses of candidate regulatory genes could provide further insights into regulatory circuits operating in *L*. *buchneri* in response to oxygen availability. Although demanding, transcriptional profiling of *L*. *buchneri* in silage would provide a more practically relevant and realistic picture of its metabolism operating in its natural habitat. Obtained results may be exploited to optimize or improve properties of silage inoculants that might be important for the ensiling process, especially the aerobic stability (oxygen consumption, oxygen conversion, hydrogen peroxide production). Genes specifying these properties could also be used as markers to screen for or develop beneficial silage inoculants.

## Supporting Information

S1 Fig Fermentation of *L*. *buchneri* CD034 grown under aerobic and anaerobic conditions. The fermentation was separated in three phases while phase 1 represents aerobic, phase 2 anaerobic and phase 3 again aerobic conditions with respect to the oxygen concentration in the gas inlet (O_2,in_). (A) Gas phase composition during fermentation as described by oxygen and carbon dioxide concentrations in the gas inlet (O_2,in_, CO_2,in_) and in the gas outlet (O_2,out_, CO_2,out_). (B) pH, dissolved oxygen concentration in the medium (dO_2_) and optical density (OD). (C) Concentration of carbohydrates glucose, xylose and arabinose and (D) concentration of organic acids lactate and acetate. Fermentations were performed in duplicates. Arrows indicate sampling time points for RNA-Seq. Parameters shown originate from fermenter 2. For fermenter 1 see Fig 1.
(TIFF)

S1 Table RNA sequencing results for *Lactobacillus buchneri* CD034 genes. The table contains information about the annotation, read count and RPKM values as determined by the ReadXplorer platform and results of the differential transcriptional analysis computed by DeSeq.
(XLSX)
