# Supplementary figures and images for: Identification of Oxygen-Responsive Transcripts in the Silage Inoculant Lactobacillus buchneri CD034 by RNA Sequencing
Source: PLoS One. 2015 Jul 31;10(7):e0134149. doi: 10.1371/journal.pone.0134149 (PMC4521753; doi:10.1371/journal.pone.0134149)

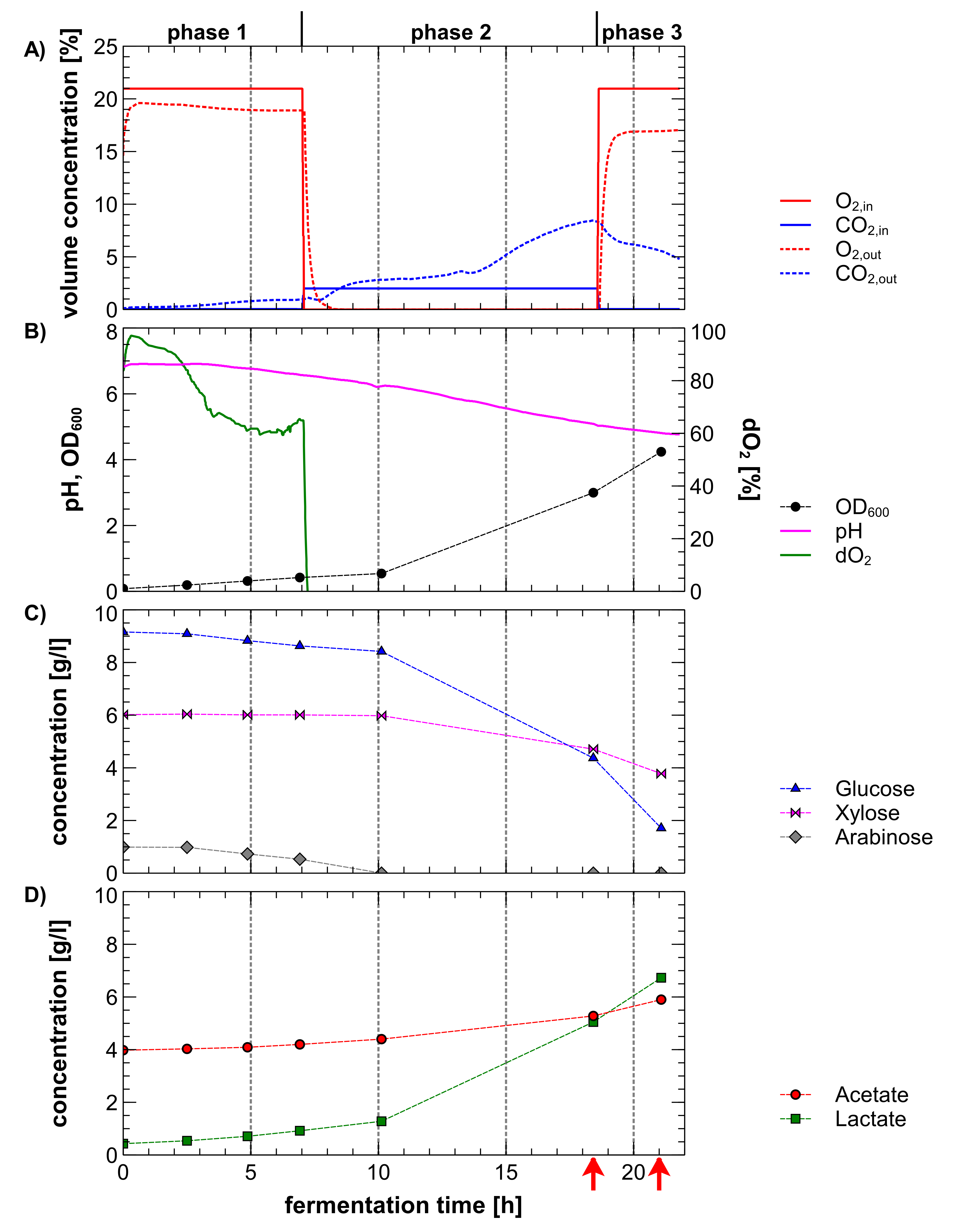

Supplement: S1 Fig — The fermentation was separated in three phases while phase 1 represents aerobic, phase 2 anaerobic and phase 3 again aerobic conditions with respect to the oxygen concentration in the gas inlet (O2,in). (A) Gas phase composition during fermentation as described by oxygen and carbon dioxide concentrations in the gas inlet (O2,in, CO2,in) and in the gas outlet (O2,out, CO2,out). (B) pH, dissolved oxygen concentration in the medium (dO2) and optical density (OD). (C) Concentration of carbohydrates glucose, xylose and arabinose and (D) concentration of organic acids lactate and acetate. Fermentations were performed in duplicates. Arrows indicate sampling time points for RNA-Seq. Parameters shown originate from fermenter 2. For fermenter 1 see Fig 1. (TIFF) [file pone.0134149.s001.tiff]
